# Severe acute hepatitis in children: Proposal to investigate *Bartonella henselae* with a multistep platform

**DOI:** 10.1371/journal.pntd.0010949

**Published:** 2022-12-15

**Authors:** Paulo Eduardo Neves Ferreira Velho, Marina Rovani Drummond

**Affiliations:** 1 Applied Research in Dermatology and Bartonella Infection Laboratory, University of Campinas, UNICAMP; Campinas, São Paulo, Brazil; 2 Division of Dermatology, Department of Medicine, UNICAMP, Campinas, São Paulo, Brazil; The University of Kansas, UNITED STATES

Dear Editor,

Severe acute hepatitis of unknown aetiology in children has been reported in 35 countries, in five of the six World Health Organization (WHO) Regions. There are 1,010 reported probable cases, including 22 deaths [1]. To confirm whether an increase in case incidence has occurred in 2022, van Beek and colleagues carried out a survey to collect data on pediatric cases of hepatitis of unknown origin (HUO) in the past 5 years. The survey showed an increase in 2022 in six of the 24 surveyed countries, with the highest number of cases being reported in the United Kingdom [2]. Similarly, Kleine and colleagues conducted a survey in 34 centers treating pediatric hepatitis in 2022. Of the surveyed centers, 22 reported no increase in pediatric cases of acute HUO from previous years, while 12 reported a suspected possible increase [3]. The surveys, however, report data until April of this year, when the case incidence was still low. From May to July, cases fulfilling WHO criteria have increased from 169 to 1,010 [3]. So, the cases of acute HUO are increasing around the world, and there is a need to understand what is causing this outbreak.

Brodin and Arditi’s study asserts that the most plausible cause of this hepatitis is an infectious agent. The authors suggest that these recently reported cases of severe acute HUO in children could be a consequence of adenovirus infection with intestinal trophism in children previously infected by Severe Acute Respiratory Syndrome Coronavirus 2 (SARS-CoV-2) responsible for the Coronavirus Disease 2019 (COVID-19) pandemic, and still carrying the virus. They recommend that an investigation be made for the presence of SARS-CoV-2 superantigens in children with severe acute cryptogenic hepatitis [4]. A report from the European Region describes that 77.3% of patients with acute HUO were 5 years old or younger; 53.5% had a positive test for adenovirus, 10.4% had a positive PCR for SARS-CoV-2, and 10.3% were coinfected with both pathogens [5]. Thus, although active infections with SARS-CoV-2 have not been identified in most of the patients reported so far, it is not unreasonable to suspect that this virus is in some way involved in the pathogenesis of the new epidemic, whether acting as superantigens related to multisystemic inflammation syndrome in children by immune activation in patients coinfected with adenovirus, or by another mechanism.

Coinfection in the human host could be the cause of the triggering of inflammation that may involve the liver of affected children, and our hypothesis agrees with the perspective in *One Health* that animal health is closely related to human health and the environment. The pandemic experience highlights that the welfare and health of people are interdependent with other living beings and the environment. Many *One Health* initiatives focus mainly on the relationship between humans and livestock or wildlife health, because several zoonotic disease pandemics and (re)emerging infectious diseases originated from these animal species. However, the role of pets, particularly dogs and cats, is often underestimated in *One Health* communications. During past decades, dogs and cats more often spend their life indoors in very close physical contact with their owners. There are several zoonotic infectious diseases that may be transmitted directly or indirectly from these animals [6]. Before the pandemic, it was estimated that there were roughly 471 million dogs and 373 million cats owned and kept as pets in the world [7]. During the SARS-CoV-2 pandemic, there was an international surge in the acquisition of cats and dogs as pets, including feral or rescued animals [8]. Homeless canines and felines were adopted from animal shelters in unprecedented numbers, and contact with pets and their ectoparasites was more extensive because of the quarantine (social containment measures imposed to limit SARS-CoV-2 spread during the COVID-19 pandemic), which enhanced the risk of infection [6]. Cats are reservoirs of *Bartonella henselae*, and many other animals, such as dogs and even humans, maintain a long-term bacteremia [9]. *B*. *henselae* is the main species of *Bartonella* genus to cause diseases in humans and is a worldwide-distributed bacterium that also causes hepatitis [10].

*B*. *henselae* is responsible for cat-scratch disease (CSD) that affects especially children and young people, in the same way as does epidemic hepatitis. In CSD, liver inflammation is the third most frequent expression, after fever and lymphadenopathy [11]. Acute/subacute liver involvement has been widely described in immunocompetent and immunodeficient *Bartonella* sp.-infected pediatric and adult patients [12], unlike adenovirus, which is only related to liver inflammation in immunodeficient patients [13]. This bacterium has already been associated with granulomatous hepatitis, bacillar angiomatosis and peliosis in the liver, and even nonspecific hepatitis [14]. We recently documented, using liquid and solid cultures and molecular tests, that *B*. *henselae* DNA was detected in blood and/or skin fragments in seven of 15 adult patients on the liver transplant waiting list diagnosed with cryptogenic hepatitis at a university hospital in Brazil. The bacteremia was able to be documented in four of these seven patients [10].

Although *B*. *henselae* can cause serious clinical manifestations and lead to death, bacteremia has been detected in blood donors as well as in immunocompetent humans, suggesting that long-standing infection by this bacterium is more prevalent in humans than is currently appreciated [15,16]. This is seemingly important in the context of a complex interaction between the bacteria and viruses. The infection by *B*. *henselae* could precede or occur after exposure to SARS-CoV-2, adenovirus, or both.

Coinfection by SARS-CoV-2, adenovirus, and *B*. *henselae* could interfere with the pathogenicity of bacteria, which, if transmitted to individuals without previous immunity to the bacteria, could potentially trigger serious liver disease, especially in children. Neu and Mainou suggest that although bacteria do not support eukaryotic virus infection, they can promote viral fitness by enhancing virion stability, promoting infection of eukaryotic cells, and increasing coinfection rates. Virus binding to bacteria can also impact bacterial biology, including bacterial adherence to eukaryotic cells [17]. The interaction of *B*. *henselae* and SARS-CoV-2 could happen in animals such as cats and dogs [18,19].

*B*. *henselae* infection serologic investigation was already proposed in HUO [20], but several studies have demonstrated the absence of a correlation between PCR detection and reactive serology. In a study conducted by Maggi and colleagues, only 30.4% of positive PCR samples were positive in serology [21]. In our previous study with 500 donors from UNICAMP’s blood bank, DNA of *Bartonella* spp. was detected in 16 donors using just one conventional PCR and only three of these were also reactive in immunofluorescence assay (IFA) [15]. It is well established in medical literature that this bacterial bloodstream infection should be better analyzed by a multistep platform with liquid and solid cultures, serology, and molecular tests [10,22–24].

Considering the presence of *B*. *henselae* worldwide, the pandemic-induced increase in human exposure to this pathogen, and bacterial and virus interaction, *B*. *henselae* infection should be investigated in children with severe acute cryptogenic hepatitis using a multistep diagnostic platform.
